# Research progress on bowel preparation regimens before colonoscopy: a narrative review

**DOI:** 10.3389/fmed.2026.1959875

**Published:** 2026-10-14

**Authors:** Xiang Liao, Yu Song, Yazhi He, Zhi Zeng, Shuang Li, Jing Ling

**Affiliations:** Endoscopy Center, Deyang People's Hospital, Deyang, Sichuan, China

**Keywords:** Boston bowel preparation scale, bowel preparation, colonoscopy, health literacy, older adults, polyethylene glycol

## Abstract

Adequate bowel preparation is the single most important determinant of colonoscopy quality, yet suboptimal preparation affects up to one-quarter of colonoscopies, leading to repeat procedures, increased health-system costs, and health disparities. This narrative review synthesizes the current evidence on bowel preparation regimens through a public health lens, organized around four interconnected dimensions: cleansing efficacy, safety, tolerability, and population-specific applicability. We define and classify preparation regimens by volume (high-volume ≥4 L, low-volume 2–<4 L, ultra-low-volume <2 L) and administration schedule (split-dose, same-day). Low-volume, split-dose formulations achieve non-inferior cleansing efficacy to high-volume 4L polyethylene glycol (PEG) regimens, with improved patient tolerability and compliance. The Boston Bowel Preparation Scale (BBPS) is the most widely adopted assessment instrument; commonly used adequate preparation is defined as total BBPS ≥6 with each segment ≥2, although definitions vary across studies. PEG-based preparations maintain the most favorable safety profile, particularly in patients with renal or cardiovascular comorbidities, while sodium phosphate carries a small but clinically significant risk of acute phosphate nephropathy. Special populations—including older adults with frailty and polypharmacy, patients with inflammatory bowel disease, and those with diabetes—require tailored preparation strategies. Emerging evidence on glucagon-like peptide-1 receptor agonists (GLP-1 RAs), linaclotide, simethicone, dietary optimization, and artificial intelligence (AI)-based bowel preparation assessment is critically evaluated. Health system implications, including the impact of inadequate preparation on resource utilization, adherence barriers linked to health literacy, and equity considerations, are examined. The review concludes that no single regimen is universally optimal; personalized preparation strategies integrating risk stratification, AI-assisted quality assessment, and evidence-based adjunctive protocols represent the most promising path toward improving colonoscopy outcomes at the population level.

## 1 Introduction

Colorectal cancer remains a leading cause of cancer-related mortality worldwide, and high-quality colonoscopy is the cornerstone of colorectal cancer screening and surveillance (1). Adequate bowel preparation is widely recognized as the single most important determinant of colonoscopy quality, directly influencing the adenoma detection rate (ADR), cecal intubation rate, and procedure-related complications (2). Despite decades of refinement in preparation protocols, up to one-quarter of colonoscopies are still associated with inadequate bowel preparation, resulting in reduced polyp and adenoma detection, screening failure, and an increased likelihood of repeat procedures (3).

From a public health perspective, suboptimal bowel preparation carries substantial downstream consequences that extend beyond the individual endoscopic encounter. Inadequate preparation leads to aborted or incomplete procedures, necessitating early repeat colonoscopies that increase health-system costs, procedural risk, and patient burden. A recent multicenter randomized trial of 665 hospitalized adults demonstrated that a very-low-volume 1L PEG-ascorbate regimen achieved higher rates of high-quality overall cleansing (46.9% vs. 35.3%–37.4%) and right-colon cleansing (40.6% vs. 29.5%–31.6%) compared with 2L and 4L PEG, with 84.2% willingness to repeat in the 1L group (4). These findings underscore the importance of optimizing preparation strategies not only for clinical efficacy but also for health-system efficiency and resource utilization.

Older adults warrant particular attention in the public health framing of bowel preparation. Age-related physiological changes—including reduced renal function, diminished thirst response, and higher prevalence of constipation—increase the risk of both inadequate preparation and preparation-related adverse events, and these risks are compounded by multimorbidity, polypharmacy, and frailty (5). Health literacy limitations disproportionately affect older and socioeconomically disadvantaged populations: a study of comprehension of written colonoscopy preparation leaflets demonstrated that low health literacy was independently associated with poorer understanding of preparation instructions (6). A prospective study of older adults further showed that lower patient activation, rather than limited health literacy *per se*, was an independent predictor of suboptimal bowel preparation quality, highlighting the importance of patient engagement strategies in this population (7). Disparities in preparation quality by education level, e-health literacy, and socioeconomic status have been documented across diverse populations, reinforcing that equity-oriented interventions—including enhanced patient education, mobile health platforms, and patient navigation—are essential components of population-level colonoscopy quality improvement (8–10).

The landscape of bowel preparation has undergone substantial transformation over the past decade, driven by a persistent tension between maximizing colonic cleansing efficacy and minimizing the volume burden and associated adverse effects that compromise patient compliance (11). This tension has catalyzed the development of a diverse armamentarium of preparation agents, yet it has also introduced substantial heterogeneity in clinical practice. A critical but often overlooked challenge is the lack of standardized assessment and reporting of bowel preparation quality, as clinicians and researchers continue to employ a heterogeneous mix of validated and nonvalidated instruments (12).

The primary aim of this narrative review is to provide a structured synthesis of the current evidence on bowel preparation regimens through a public health lens, organized around four interconnected dimensions: cleansing efficacy, safety, tolerability, and population-specific applicability. Each dimension is evaluated against the current reference regimen of split-dose 4L PEG, while explicitly addressing the public health implications of regimen choice—including health-system resource utilization, equity in access to high-quality colonoscopy, adherence barriers in vulnerable populations, and the growing challenge of preparing an aging, multimorbid population for colorectal cancer screening.

## 2 Methods

This narrative review was conducted following a structured methodology to enhance transparency and reproducibility. A systematic literature search was performed across PubMed, Embase, the Cochrane Library, and Web of Science for English-language articles published between January 2018 and August 2026. Search terms included combinations of “bowel preparation,” “colonoscopy,” “polyethylene glycol,” “oral sulfate,” “sodium phosphate,” “cleansing quality,” “split-dose,” “Boston Bowel Preparation Scale,” “patient tolerability,” “older adults,” “frailty,” “health literacy,” “GLP-1 receptor agonist,” “linaclotide,” “simethicone,” “low-residue diet,” “inflammatory bowel disease,” and “artificial intelligence.” Boolean operators were used to combine terms within each conceptual domain and across domains.

Study selection was guided by the following inclusion criteria: (1) randomized controlled trials (RCTs), systematic reviews, meta-analyses, and clinical practice guidelines addressing bowel preparation for colonoscopy; (2) studies reporting on at least one of the four evaluation dimensions (efficacy, safety, tolerability, or population-specific considerations); and (3) studies published in peer-reviewed journals. Conference abstracts, editorials, and studies focusing exclusively on CT colonography or surgical bowel preparation were excluded. Evidence was prioritized hierarchically: clinical practice guidelines from major international societies (ESGE, US Multi-Society Task Force) were given the highest weight, followed by systematic reviews and meta-analyses, individual RCTs, and large observational studies. Additional articles were identified through manual review of reference lists from included studies.

After deduplication and title/abstract screening, 60 full-text articles were reviewed and 41 were selected for final inclusion based on the evidence prioritization hierarchy. Of the 41 included studies, 39 were identified through the database search; the remaining 2 (published 2012 and 2014) were retained as foundational public-health evidence as described above. The included studies comprise clinical practice guidelines (*n* = 4), systematic reviews and meta-analyses (*n* = 18), individual RCTs (*n* = 14), and large observational studies (*n* = 5). Data were extracted on study design, population, preparation regimen (agent, volume, dosing schedule), cleansing assessment scale, primary and secondary outcomes, and safety and tolerability endpoints. Given the heterogeneity of study designs, populations, and outcome measures, a narrative synthesis approach was adopted, with evidence organized within the four-dimensional evaluation framework and synthesized according to the public health themes identified *a priori*.

## 3 Classification and terminology of bowel preparation regimens

Establishing a standardized classification system for bowel preparation regimens is essential for meaningful comparison across studies and for evidence-based clinical decision-making. The European Society of Gastrointestinal Endoscopy (ESGE) 2019 guideline provides the most comprehensive framework for categorizing preparation agents, recommending the use of high-volume or low-volume PEG-based regimens as well as clinically validated non-PEG-based agents for routine bowel preparation. The ESGE guideline also strongly recommends split-dose bowel preparation for elective colonoscopy, with a same-day regimen as an acceptable alternative for afternoon procedures (13).

### 3.1 Volume-based classification

Bowel preparation regimens can be systematically classified by the total volume of purgative solution ingested.

#### 3.1.1 High-volume regimens (≥4 L)

The traditional 4L PEG electrolyte lavage solution (PEG-ELS) is a non-absorbable, iso-osmotic formulation that remains the historical reference standard. However, the large volume requirement is the primary barrier to patient compliance (14).

#### 3.1.2 Low-volume regimens (2–<4 L)

The 2L PEG-ascorbate (PEG-Asc) formulation, which combines the osmotic effect of PEG with the adjuvant action of ascorbic acid, has been the most extensively studied low-volume alternative. A systematic review and meta-analysis of 17 RCTs including 7,528 patients confirmed that low-volume, split-dose regimens achieve non-inferior adequacy of bowel cleansing compared with high-volume split-dose regimens (86.1% vs. 87.4%; RR 1.00, 95% CI 0.98–1.02), with superior tolerability and willingness to repeat (11).

#### 3.1.3 Ultra-low-volume regimens (<2 L)

The 1L PEG-Asc formulation represents the most recent advance, achieving high rates of adequate preparation in both clinical trials and real-world settings. A large real-world multicenter observational study of over 13,000 patients demonstrated that 1L PEG-Asc delivered high levels of adequate and high-quality bowel cleansing (15). In elderly patients aged 65–85 years, 1L PEG-Asc was as effective as 2L PEG-Asc, with higher rates of perfect overall preparation (40.2% vs. 25.0% by Harefield Cleansing Scale) and superior right colon cleansing quality, without significant differences in safety profiles (16). Among hospitalized adults, 1L PEG-Asc achieved adequate overall cleansing in 82.0% of patients, with a higher rate of high-quality cleansing than 2L or 4L PEG (4).

### 3.2 Administration schedule

#### 3.2.1 Split-dose regimen

Half of the preparation volume is ingested the evening before colonoscopy and the remaining half on the morning of the procedure. Split-dose administration is the single most important modifiable factor for optimizing cleansing quality. The ESGE recommends that the last dose of bowel preparation be started within 5 h and completed at least 2 h before colonoscopy (13). The US Multi-Society Task Force consensus likewise recommends split-dose bowel preparation as the standard of care, with a target of >90% adequate preparation rate per endoscopist and per endoscopy unit (1). A pragmatic RCT demonstrated that mandatory split-dose preparation was superior to optional split-dose for early morning colonoscopies (89.9% vs. 78.9% adequate BBPS), establishing that split dosing should be prescribed as a requirement rather than a choice (17).

#### 3.2.2 Same-day regimen

The entire preparation is consumed on the morning of the procedure. This approach is an acceptable alternative for afternoon colonoscopies. In patients with gastrectomy undergoing afternoon colonoscopy, same-day and split-dose PEG achieved comparable cleansing success (92.6% vs. 95.9%) (18). The 2025 US Multi-Society Task Force consensus suggests that same-day regimens are acceptable for afternoon procedures but may be inferior for morning colonoscopies (1).

### 3.3 Non-PEG alternatives

Non-PEG alternatives, including oral sulfate solution (OSS) and sodium phosphate (NaP) tablets, offer distinct advantages in palatability and reduced fluid intake. A systematic review of 13 RCTs including 2,773 patients found that NaP tablets achieved cleansing quality comparable to PEG lavage (RR 1.02, 95% CI 0.96–1.08), with significantly lower rates of nausea (RR 0.67, 95% CI 0.58–0.76) and higher willingness to repeat (RR 1.52, 95% CI 1.28–1.80) (19). However, NaP was associated with greater decreases in serum potassium and calcium and a substantial increase in serum phosphorus (mean difference 4.51 mg/dL), underscoring the need for electrolyte monitoring. The contraindication of NaP in patients with renal impairment, congestive heart failure, and advanced age is well established (19, 20).

## 4 Bowel preparation quality assessment scales

Validated bowel preparation quality scales are essential for standardized assessment of cleansing efficacy and for meaningful comparison across clinical trials. However, the heterogeneity of scales used across studies remains a significant barrier to evidence synthesis (12).

### 4.1 Boston bowel preparation scale (BBPS)

The BBPS assigns segment-specific scores (0–3) to the right, transverse, and left colon after all washing and suctioning, yielding a total score of 0–9. Among the available instruments, the BBPS has the strongest evidence base for inter-observer reliability and is the most widely adopted scale in clinical trials and practice. The ESGE defines adequate bowel preparation as BBPS ≥6 (13), and the most commonly used threshold in clinical trials is total BBPS ≥6 with each segment ≥2, although individual studies have employed varying definitions (13, 21, 22). A dose-response relationship between higher BBPS scores and improved ADR has been consistently observed. However, the BBPS should be considered the most widely validated and adopted instrument in current practice rather than an absolute “reference standard,” as its performance depends on endoscopist training and the timing of assessment (pre- vs. post-washing). Several studies have employed the term “reference standard” loosely; we consider it more accurate to describe the BBPS as the most extensively validated assessment tool currently available, while acknowledging that alternative scales may be more suitable depending on the clinical or research context.

### 4.2 Ottawa bowel preparation scale (OBPS)

The OBPS evaluates cleanliness and fluid volume in each colonic segment using a composite score (range 0–14, where lower scores indicate better preparation). Adequate preparation on the OBPS is typically defined as ≤7 (21). The OBPS has the advantage of separately quantifying residual fluid, which may be clinically relevant for certain procedural indications. However, its greater complexity and less intuitive scoring direction have limited its adoption relative to the BBPS.

### 4.3 Harefield cleansing scale (HCS)

The HCS incorporates an overall adequacy rating (grades A–D) in addition to segmental assessment. Successful preparation is defined as grade A or B. The HCS offers the advantage of explicitly classifying preparations as “successful” or “unsuccessful,” which aligns well with clinical decision-making regarding follow-up intervals. The HCS has demonstrated comparable performance to the BBPS in head-to-head validation studies.

### 4.4 Other validated instruments

The Aronchick Scale, an older instrument using a 1–5 global rating (excellent to inadequate), remains in use particularly in older trials, though its global rather than segmental assessment limits its clinical utility. The Colon Endoscopic Bubble Scale (CEBuS) addresses the specific problem of intraluminal bubbles that impair mucosal visualization. Taveira et al. demonstrated that CEBuS provides almost perfect intraobserver reliability (kappa 0.86) and high interobserver agreement (ICC 0.90), independent of endoscopic experience (23). This scale complements the BBPS by quantifying a distinct aspect of preparation quality not captured by conventional cleansing scales.

### 4.5 Comparative evidence

Direct comparisons among scales are limited by the absence of a true gold standard for bowel preparation quality. The BBPS and OBPS have been directly compared in validation studies, with the BBPS generally demonstrating higher inter-observer agreement. The ESGE quality improvement initiative for IBD colonoscopy recommends using any of the three validated scales (BBPS, OBPS, or Aronchick), defining adequate preparation as BBPS ≥6, OBPS ≤7, or Aronchick excellent/good/fair (21). A key limitation across the evidence base is that studies use different definitions of “adequate” preparation even within the same scale, complicating cross-study comparisons. The ongoing lack of a universally accepted definition of adequate preparation represents a persistent barrier to evidence synthesis. Table 1 summarizes these properties side by side.

**Table 1:** Comparative characteristics of validated bowel preparation quality scales.

Scale	Segmental scoring	Timing relative to washing	Score range (direction)	Validated adequacy threshold	Key advantages	Key limitations
Aronchick Scale	None; single global grade	Before	1–5 (higher worse)	None defined	Rapid and simple; historically the most used instrument	No segmental information; weak distal-colon reliability; no outcome validation
Ottawa Bowel Preparation Scale	3 segments + separate global fluid score	Before	0–14 (lower better)	≥8 indicates inadequate (sensitivity 100%, specificity 91%)	Segmental plus fluid assessment; high interobserver reliability	Reverse scoring direction; validated mainly against the Aronchick Scale; fluid score confounded by water exchange
Boston Bowel Preparation Scale	3 segments	After	0–9 (higher better)	Total ≥6 AND every segment ≥2	Validated against polyp/adenoma detection and surveillance intervals; strongest evidence base	Post-washing score mixes intrinsic purgative efficacy with endoscopist washing effort
Harefield Cleansing Scale	5 segments	After	0–20 (higher better)	Grades A–B successful; C–D unsuccessful	Built-in binary verdict; test–retest and internal-consistency data	Five-segment grid is cumbersome; weaker left-colon reliability; validated only against the Aronchick Scale
Chicago Bowel Preparation Scale	3 segments + separate fluid score	Before (fluid) and after (mucosa)	0–36 (higher better); fluid 0–3	None defined	Captures both pre-wash soiling and cleaning effort	Complex; only two validation raters; no correlation with clinical outcomes
Colon Endoscopic Bubble Scale	Whole-colon bubble burden	During inspection	Bubble grades	–	Quantifies intraluminal bubbles missed by stool-cleansing scales; almost perfect intraobserver reliability	Narrow construct (foam only); does not grade solid or liquid residue

## 5 Efficacy of bowel preparation regimens

### 5.1 Head-to-head comparisons across regimen classes

The central efficacy question in bowel preparation research has been whether low-volume and ultra-low-volume regimens can match the cleansing efficacy of high-volume 4L PEG. The evidence from systematic reviews and meta-analyses consistently demonstrates non-inferiority. The meta-analysis by Spadaccini et al., synthesizing 17 RCTs, provided the most comprehensive answer: low-volume split-dose regimens achieved comparable adequate cleansing rates to high-volume regimens, with minimal heterogeneity (I^2^ = 17%) and no significant difference in ADR (RR 0.96, 95% CI 0.87–1.08) (11). The overall quality of evidence was rated as moderate using the GRADE framework.

The recently published multicenter RCT by Frazzoni et al., which randomized 665 hospitalized adults to 1L, 2L, and 4L PEG-ascorbate, provides important new evidence (4). Adequate overall cleansing occurred in 82.0%, 78.0%, and 78.5% of patients, respectively. Critically, 1L PEG-Asc yielded higher rates of high-quality overall cleansing (46.9% vs. 35.3%–37.4%) and high-quality right-colon cleansing (40.6% vs. 29.5%–31.6%), with the highest willingness to repeat (84.2%). These results challenge the assumption that higher-volume preparations necessarily produce better cleansing in hospitalized patients and have direct implications for health-system resource allocation and patient-centered care.

Several comparisons in specific populations warrant attention. In hospitalized children, low-volume PEG (40 mL/kg) was non-inferior to 60 mL/kg for whole-colon and segmental cleansing, with higher patient satisfaction (24). In patients with gastrectomy, same-day and split-dose PEG achieved comparable cleansing success (92.6% vs. 95.9%), suggesting that dosing schedule can be tailored to individual patient logistics without compromising efficacy (18).

### 5.2 Predictive models for inadequate preparation

Predictive models and risk scores have been developed to identify patients at high risk of inadequate preparation. Fostier et al. reviewed the evidence for these models and noted that while they perform reasonably well in derivation cohorts, external validation remains limited and their integration into routine clinical workflows has been slow (25). The most consistent predictors of inadequate preparation across studies include constipation, diabetes mellitus, opioid use, prior inadequate preparation, and afternoon colonoscopy scheduling. The limited external validation of these prediction models in diverse populations—particularly in low- and middle-income countries and among patients with low health literacy—represents a critical evidence gap that constrains their clinical utility.

## 6 Safety and tolerability

### 6.1 Electrolyte and renal safety across preparation classes

The safety profiles of bowel preparation agents diverge markedly across classes. PEG-based preparations have the most favorable safety profile due to their iso-osmotic formulation, ensuring that net fluid and electrolyte shifts are minimal. This osmotic stability is particularly critical in patients with chronic kidney disease (CKD) and congestive heart failure, who are at heightened risk of volume overload and electrolyte disturbances during the purgative process. The ESGE guideline recommends PEG-based preparations as the preferred choice in patients with renal or cardiovascular comorbidities (13), a recommendation supported by nationwide cohort data demonstrating that PEG formulations are associated with lower rates of post-colonoscopy systemic adverse events compared with non-PEG alternatives (26). However, the optimal volume of PEG in this high-risk population remains an area of uncertainty. While ultra-low-volume PEG regimens reduce total fluid intake and may theoretically lower the risk of volume overload, dedicated prospective studies specifically enrolling patients with CKD stage 3b–5 or decompensated heart failure are lacking. Available evidence on ultra-low-volume regimens derives predominantly from general populations without stratification by renal function or cardiac status (4, 15), limiting the extrapolation of these findings to volume-sensitive patients. Furthermore, the growing use of diuretics, renin-angiotensin system inhibitors, and sodium-glucose cotransporter-2 inhibitors in patients with CKD and heart failure raises additional concerns about preparation-related electrolyte perturbations, yet no prospective studies have systematically evaluated these drug–preparation interactions.

At the opposite end of the safety spectrum, NaP-based preparations carry a small but clinically significant risk of acute phosphate nephropathy, which prompted the United States Food and Drug Administration to issue a boxed warning. This risk is substantially amplified in patients with CKD, congestive heart failure, and advanced age, in whom NaP is strictly contraindicated due to the compounded risk of electrolyte shifts and volume-related adverse events (13, 19). The electrolyte disturbances associated with NaP—including hyperphosphatemia, hypokalemia, and hypocalcemia—can precipitate arrhythmias and acute kidney injury, particularly in patients with impaired renal perfusion or cardiac dysfunction. Lee et al. demonstrated that bowel preparation for colonoscopy may decrease serum testosterone levels in Korean men, highlighting that preparation-related physiological perturbations extend beyond the gastrointestinal and renal systems (27). Tong et al., in an observational study of type 2 diabetes patients, found that GLP-1 RA liraglutide and DPP-4 inhibitor sitagliptin did not significantly increase the incidence of inadequate bowel cleaning overall, although GLP-1 RA use was associated with higher rates of inadequate preparation in the subgroup with diabetic peripheral neuropathy (61.3% vs. 32.1%–32.8%) (28). Sulfate-based preparations occupy an intermediate safety position; while oral sulfate solution has demonstrated acceptable safety in selected populations including patients with IBD (20), its use in patients with significant renal or cardiac impairment requires caution, and dedicated safety data in CKD and heart failure populations remain scarce. Notably, despite the clinical importance of volume-sensitive populations, the current evidence base for bowel preparation safety in CKD and heart failure patients relies almost entirely on subgroup analyses and extrapolation from general-population studies, rather than dedicated prospective investigations with prespecified renal and cardiac safety endpoints. There is a pressing need for comparative-effectiveness trials that stratify patients by renal function and cardiac status, and that quantify the relationship between preparation volume, electrolyte shifts, and clinically meaningful renal or cardiac outcomes.

### 6.2 Tolerability and patient-reported outcomes

Patient tolerability is a critical determinant of preparation compliance and, consequently, of colonoscopy quality and health-system efficiency. Across preparation classes, the most commonly reported adverse symptoms include nausea, bloating, abdominal pain, and vomiting. Taste acceptability consistently ranks as the most important factor influencing patient willingness to repeat the preparation. A meta-analysis of 11 RCTs of linaclotide combined with PEG versus PEG alone demonstrated that linaclotide reduced abdominal pain, bloating, nausea, and sleep disturbance while increasing willingness to repeat (29). Furthermore, 2L PEG plus linaclotide was non-inferior to 3L PEG alone, supporting its role in low-volume preparation strategies. Another systematic review of 8 RCTs including 3,591 patients confirmed that linaclotide plus PEG improved preparation quality (MD 0.31; 95% CI [0.08, 0.54]) and willingness to repeat (RR 1.16; 95% CI [1.09, 1.23]), with reduced adverse events (30). Subgroup analyses suggested particular benefit in patients with constipation.

### 6.3 Adherence-enhancing strategies

Enhanced patient education has emerged as an effective, low-cost strategy to improve preparation quality independent of the specific agent used. Lam et al. demonstrated in a population-based colorectal cancer screening program that nurse-led reinforced education delivered through a mobile messenger platform significantly improved bowel preparation quality compared to standard written instructions (9). From a public health perspective, these interventions are particularly relevant because they address adherence barriers that are disproportionately concentrated in populations with lower health literacy and fewer healthcare resources. The US Multi-Society Task Force recommends the adjunctive use of oral simethicone, routine tracking of adequate preparation rates at the endoscopist and unit level (target >90%), and limiting dietary restrictions to the day before colonoscopy (1).

## 7 Special populations: multimorbidity, frailty, and equity considerations

### 7.1 Older adults, frailty, and polypharmacy

Patients aged 70 years and older represent a growing proportion of the colonoscopy population and present unique challenges. Age-related physiological changes—including reduced renal function, diminished thirst response, and higher prevalence of constipation—increase the risk of both inadequate preparation and preparation-related adverse events, and these risks are compounded by multimorbidity and polypharmacy (5). PEG-based preparations are recommended in this population due to their favorable safety profile. A randomized study demonstrated that 1L PEG-Asc was as effective as 2L PEG-Asc in patients aged 65–85 years, with higher rates of perfect preparation (40.2% vs. 25.0%) and superior right colon cleansing without increased adverse events, suggesting that ultra-low-volume regimens may be particularly suitable for elderly patients with comorbidities (16).

Frailty assessment, rather than chronological age alone, is increasingly recognized as a more informative guide to preparation selection. Older adults with frailty, cognitive impairment, and polypharmacy are at elevated risk of preparation-related adverse events and of inadequate preparation due to challenges in adhering to complex preparation instructions. Low health literacy is independently associated with poorer comprehension of written preparation instructions (6). Lower patient activation has been identified as an independent predictor of suboptimal preparation quality among older adults undergoing colonoscopy (7). A large prospective study of 9,618 patients in China demonstrated that low perioperative e-health literacy was an independent risk factor for inadequate bowel preparation, with inadequate preparation predominantly affecting the right and proximal colon (8).

### 7.2 Inflammatory bowel disease

IBD patients require mucosal-sparing preparation strategies to avoid preparation-induced artifact that can confound assessment of disease activity. A systematic review and network meta-analysis of 960 patients from 7 RCTs demonstrated that various bowel preparations had similar effectiveness for colonoscopy preparation in IBD patients, with low-volume preparations demonstrating better compliance, tolerance, and acceptance (31). The ESGE quality improvement initiative for colonoscopy in IBD emphasizes that preparation quality assessment in this population requires disease-specific benchmarks beyond those used in the general screening population (21).

### 7.3 Diabetes and GLP-1 receptor agonists

Patients with diabetes face the dual challenge of glycemic management during the fasting and purging period and an increased risk of inadequate preparation due to diabetes-related gastrointestinal dysmotility (32). The emerging use of GLP-1 RAs has introduced a new and clinically important consideration. A recent systematic review and meta-analysis of six studies found no significant difference in the pooled rate of inadequate bowel preparation events between GLP-1 RA users and controls (OR 1.00, 95% CI 0.73–1.37), although mean BBPS scores were significantly lower in the GLP-1 RA group (mean difference −0.36, 95% CI −0.44 to −0.28) (33). A large matched regional cohort study of 503 patients in each arm found that GLP-1 RA use was associated with increased odds of inadequate bowel preparation (OR 2.6, 95% CI 1.9–3.7) and a higher rate of repeat procedures (OR 3.0, 95% CI 1.4–6.5) (34). The evidence, though based predominantly on retrospective designs with potential confounding by diabetes status, suggests that GLP-1 RA use may increase the risk of inadequate preparation, particularly in patients with normal to overweight BMI (35). Current practice is evolving toward individualized decision-making—considering factors such as BMI, diabetes duration, and prior preparation quality—rather than universal withholding of GLP-1 RAs before colonoscopy, though evidence-based guidelines remain under development.

## 8 Optimizing the preparation protocol: diet, adjunctive agents, and emerging strategies

### 8.1 Dietary optimization

Dietary modification is a critical yet underappreciated component of the bowel preparation protocol with implications for patient adherence and health equity. A meta-analysis of randomized controlled trials demonstrated that a low-residue diet (LRD) is non-inferior to a clear-liquid diet (CLD) for bowel preparation before colonoscopy, with significantly improved tolerability and willingness to repeat the preparation (22). A more recent meta-analysis comparing 1-day versus 3-day LRD demonstrated that 1-day LRD was comparable to 3-day LRD for adequate bowel preparation (OR 0.89, 95% CI 0.65–1.21) with significantly higher tolerability (OR 1.64, 95% CI 1.13–2.39), supporting a more patient-centered approach to dietary restrictions (36). The 2025 US Multi-Society Task Force consensus recommends limiting dietary restrictions to the day before colonoscopy, relying on either clear liquids or low-fiber/low-residue diets for the early and midday meals (1). From a public health perspective, less restrictive dietary regimens may improve colonoscopy uptake and completion rates among populations that face barriers to adherence.

### 8.2 Simethicone as an adjunctive agent

Simethicone, a surfactant that reduces the surface tension of gas bubbles, is the most extensively studied adjunctive agent for bowel preparation. A systematic review and meta-analysis of 16 RCTs including 5,630 patients found that PEG with simethicone improved colon cleansing compared with PEG alone (OR 1.48, 95% CI 1.11–1.97), with a significant reduction in abdominal bloating (OR 2.33, 95% CI 1.70–3.20) (37). Notably, the cleansing benefit was observed primarily with single-dose PEG regimens and was not statistically significant when split-dose PEG was used, suggesting that simethicone may be most useful in patients who cannot tolerate split dosing. The US Multi-Society Task Force suggests the adjunctive use of oral simethicone for bowel preparation before colonoscopy (1).

### 8.3 Linaclotide as a prokinetic adjunct

Linaclotide, a guanylyl cyclase-C agonist, has emerged as a promising adjunct to PEG-based bowel preparation, particularly in patients with constipation. A meta-analysis of 11 RCTs demonstrated that linaclotide combined with PEG provided comparable overall bowel cleansing to PEG alone while reducing adverse events and improving patient acceptance (29). Critically, 2L PEG plus linaclotide was non-inferior to 3L PEG alone, supporting its role in volume-reduction strategies. Subgroup analyses showed that 3L PEG plus linaclotide was superior to 3L PEG alone regarding adequate preparation (RR 1.11, 95% CI 1.01–1.23) and total BBPS score (MD 0.44, 95% CI 0.04–0.85) (29). A multicenter endoscopist-blinded RCT (Apple trial) randomized 1,464 patients to 1L PEG-Asc plus linaclotide versus 1L PEG-Asc plus senna and found that the linaclotide regimen was superior for adequate bowel preparation (92% vs. 86%), with the greatest benefit in high-risk patients (94% vs. 86%) (38). These findings position linaclotide as a particularly valuable adjunct for patients at high risk of inadequate preparation, including those with constipation.

### 8.4 GLP-1 receptor agonists and preparation adequacy

The impact of GLP-1 RAs on bowel preparation quality has become a rapidly evolving area of clinical investigation. A systematic review and meta-analysis of six studies by Ahmed et al. found no significant difference in inadequate bowel preparation rates between GLP-1 RA users and controls (OR 1.00, 95% CI 0.73–1.37, I^2^ = 33%), although mean BBPS scores were significantly lower in the GLP-1 RA group (MD –0.36, 95% CI −0.44 to −0.28) (33). In contrast, a large matched cohort study of 6,235 patients found that patients using GLP-1 RAs were significantly more likely to have inadequate bowel preparation, even after accounting for comorbidities (39). A separate matched regional cohort study of 503 GLP-1 RA users and 503 controls demonstrated that GLP-1 RA use was associated with increased odds of inadequate preparation (OR 2.6, 95% CI 1.9–3.7) and repeat procedures (OR 3.0, 95% CI 1.4–6.5) (34). An important finding from a large cohort of 51,268 patients is that the effect of GLP-1 RAs on inadequate preparation may be BMI-dependent: increased risk was observed in normal and overweight patients (OR 1.66, 95% CI 1.37–2.02) but attenuated in patients with class III obesity (35). The conflicting evidence likely reflects confounding by the underlying conditions for which GLP-1 RAs are prescribed (diabetes, obesity), heterogeneity in study design and population, and variation in preparation protocols. Current evidence does not support universal withholding of GLP-1 RAs before colonoscopy; however, individualized strategies—including consideration of BMI, diabetes status, and prior preparation quality—should guide clinical decision-making.

## 9 Artificial intelligence for bowel preparation assessment

AI-based bowel preparation assessment represents one of the most promising frontiers in colonoscopy quality improvement; however, current claims about AI performance must be critically evaluated. Deep learning-based systems can provide real-time, objective BBPS-equivalent scoring during the withdrawal phase of colonoscopy, with the potential to reduce inter-observer variability and standardize quality reporting across endoscopists and institutions.

Feng et al. developed and validated ViENDO, a three-dimensional deep learning-based system for assessing bowel preparation on full-length colonoscopy videos (40). The system achieved an AUC of 0.98 and accuracy of 95.2% for determining inadequate preparation in video clips, with 93.8% accuracy in an internal test set and 91.7% in an external dataset. In a human-machine contest, ViENDO’s accuracy was slightly superior to most endoscopists—though importantly, this difference did not reach statistical significance. These results suggest that AI systems can already perform at a level approaching that of experienced clinicians, but they do not yet demonstrate clear superiority.

Several critical caveats must guide the interpretation of AI evidence for bowel preparation assessment. First, studies of AI for general polyp detection (CADe) are distinct from studies of AI for bowel preparation quality assessment and should not be conflated. AI-based preparation assessment and AI-based polyp detection address fundamentally different clinical tasks, and evidence from one domain does not validate performance in the other. Second, current AI systems for preparation assessment have been validated primarily in single-center or limited multicenter settings with selected patient populations, and their generalizability to diverse real-world clinical environments remains incompletely established. Third, the development of large, multicenter datasets such as REAL-Colon (41) is critical for advancing the field, but dataset availability alone does not equate to clinical validation of specific AI applications. Fourth, regulatory approval pathways for AI-based medical devices are still evolving, and the cost-effectiveness of AI implementation in routine colonoscopy practice has not been demonstrated. Premature claims that AI systems are “equivalent to expert assessment” should be avoided.

## 10 Public health implications: screening effectiveness, repeat procedures, and resource utilization

The public health burden of inadequate bowel preparation extends beyond the individual patient encounter to affect population-level colorectal cancer screening outcomes, health-system costs, and equity in access to high-quality care. Inadequate preparation leads to aborted or incomplete procedures, necessitating early repeat colonoscopies that consume endoscopy unit capacity, increase procedural risk, and impose additional out-of-pocket costs on patients. The US Multi-Society Task Force has established a target of >90% adequate preparation rate per endoscopist and per endoscopy unit, with routine tracking as a quality metric (1).

From an equity perspective, the burden of inadequate preparation is not evenly distributed. Health literacy limitations disproportionately affect socioeconomically disadvantaged and older populations, and low health literacy is associated with poorer comprehension of preparation instructions and lower preparation quality (6, 8). Patient navigation programs combined with tailored educational interventions have been shown to increase colorectal cancer screening completion among low-income and minority populations who did not attend scheduled screening colonoscopy (10). These findings support a public health approach in which preparation optimization is viewed not merely as a technical clinical task but as a health equity intervention.

The growing population of older adults with multimorbidity and polypharmacy presents an escalating public health challenge for colorectal cancer screening programs. Age-related physiological changes increase the risk of both inadequate preparation and preparation-related adverse events, and the complexity of bowel preparation regimens may be particularly burdensome for patients with cognitive impairment, frailty, or limited social support (5). The development of ultra-low-volume regimens, simplified dietary protocols, and enhanced patient education strategies tailored to the needs of older and vulnerable populations should be prioritized as a public health research agenda.

## 11 Conclusion

The evidence synthesized in this review, evaluated through the four-dimensional framework of efficacy, safety, tolerability, and population-specific applicability, supports the following conclusions. First, low-volume and ultra-low-volume split-dose PEG and sulfate-based regimens achieve non-inferior cleansing efficacy to high-volume 4L PEG while offering improved patient tolerability, higher willingness to repeat, and potential health-system benefits through reduced repeat procedure rates. Second, PEG-based preparations maintain the most favorable safety profile across populations, while NaP preparations require careful patient selection and electrolyte monitoring. Third, the BBPS is the most widely validated assessment instrument in current practice; commonly used adequate preparation is defined as total BBPS ≥6 with each segment ≥2, although heterogeneity in definitions across studies remains a barrier to evidence synthesis. Fourth, special populations—particularly older adults with frailty and polypharmacy, patients with IBD, and patients using GLP-1 RAs—require individualized preparation strategies that integrate patient-specific risk profiles. Fifth, adjunctive agents including simethicone and linaclotide, dietary optimization with LRD, and AI-assisted quality assessment represent evidence-based advances that can improve preparation outcomes, though their incremental benefit is most pronounced when baseline protocols are suboptimal.

Several key knowledge gaps and research priorities warrant emphasis. First, the optimal bowel preparation strategy for patients with CKD and heart failure—populations at heightened risk of volume overload and electrolyte disturbances—remains inadequately defined. Current best practice recommendations favor PEG-based preparations in these groups, but evidence derives largely from extrapolation rather than dedicated trials that stratify by renal function or cardiac status and include prespecified renal and cardiac safety endpoints. Comparative-effectiveness studies quantifying the relationship between preparation volume, electrolyte shifts, and clinically meaningful outcomes in volume-sensitive patients are urgently needed. Second, the impact of GLP-1 RAs on bowel preparation quality requires further prospective investigation, as current evidence is limited by retrospective designs and potential confounding. Third, standardized AI validation protocols for bowel preparation assessment have not been established, and cost-effectiveness data are lacking. Fourth, large-scale comparative-effectiveness trials in low- and middle-income countries are absent, limiting the generalizability of current evidence. Fifth, long-term data linking preparation quality to post-colonoscopy colorectal cancer outcomes remain limited. Looking forward, the integration of personalized preparation strategies—combining validated risk stratification (including renal and cardiac risk factors), AI-assisted quality assessment, and evidence-based adjunctive protocols—represents the most promising path toward closing the persistent gap between preparation quality and colorectal cancer prevention outcomes at the population level. Future research should prioritize pragmatic comparative-effectiveness trials that reflect real-world clinical practice and include dedicated safety endpoints for vulnerable populations; the development and external validation of point-of-care risk stratification tools that incorporate comorbidity burden; and rigorous multicenter prospective evaluation of AI-assisted quality assessment with attention to equity, volume optimization in high-risk groups, and cost-effectiveness.
